# Janus Kinase Signaling: Oncogenic Criminal of Lymphoid Cancers

**DOI:** 10.3390/cancers13205147

**Published:** 2021-10-14

**Authors:** Boheng Li, Qin Wan, Zhubo Li, Wee-Joo Chng

**Affiliations:** 1 College of Pharmaceutical Sciences, Southwest University, Chongqing 400715, China; liboheng1023@swu.edu.cn or wqicry961029@email.swu.edu.cn (Q.W.); 2 Department of Haematology-Oncology, National University Cancer Institute of Singapore, Singapore 119074, Singapore; 3 Cancer Science Institute of Singapore, National University of Singapore, Singapore 117599, Singapore; 4 Department of Medicine, Yong Loo Lin School of Medicine, National University of Singapore, Singapore 119228, Singapore

**Keywords:** JAK/STAT, lymphoma, myeloma, inhibitor

## Abstract

**Simple Summary:**

Janus kinases (JAKs) are transmembrane receptors that pass signals from extracellular ligands to downstream. Increasing evidence has suggested that JAK family aberrations promote lymphoid cancer pathogenesis and progression through mediating gene expression via the JAK/STAT pathway or noncanonical JAK signaling. We are here to review how canonical JAK/STAT and noncanonical JAK signalings are represented and deregulated in lymphoid malignancies and how to target JAK for therapeutic purposes.

**Abstract:**

The Janus kinase (JAK) family are known to respond to extracellular cytokine stimuli and to phosphorylate and activate signal transducers and activators of transcription (STAT), thereby modulating gene expression profiles. Recent studies have highlighted JAK abnormality in inducing over-activation of the JAK/STAT pathway, and that the cytoplasmic JAK tyrosine kinases may also have a nuclear role. A couple of anti-JAK therapeutics have been developed, which effectively harness lymphoid cancer cells. Here we discuss mutations and fusions leading to JAK deregulations, how upstream nodes drive JAK expression, how classical JAK/STAT pathways are represented in lymphoid malignancies and the noncanonical and nuclear role of JAKs. We also summarize JAK inhibition therapeutics applied alone or synergized with other drugs in treating lymphoid malignancies.

## 1. Introduction

**Figure 1:**
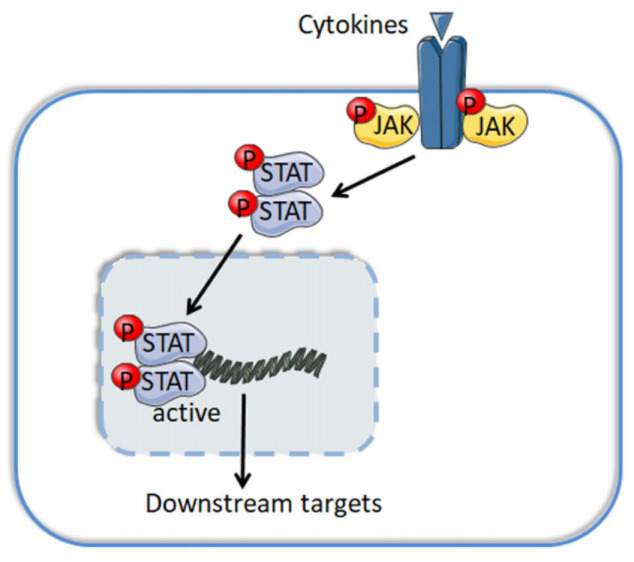
Schematic model indicating the JAK/STAT pathway.

Lymphoid cancers are lethal malignancies, which include lymphomas, myeloma and lymphoid leukemias. The Janus kinase (JAK) family comprises four members: JAK1, JAK2, JAK3 and TYK2. Structurally, all JAKs contain a FERM domain, a SH2 domain, a pseudokinase domain and a catalytic kinase domain. The JAK tyrosine kinases are mainly located in the cytoplasm and transmit signals from cytokines and their γ-chain receptors to signal transducers and activators of transcription (STAT), and the phosphorylated, dimerized and activated STAT then binds to chromosome and trans-regulates gene expression (Figure 1). There are seven members in the mammalian STAT family: STAT1, STAT2, STAT3, STAT4, STAT5A, STAT5B and STAT6 [1]. The JAK/STAT pathway is evolutionarily conserved and directly affects developmental hematopoiesis and oncogenic proliferation and migration. JAK deregulates, either by mutations and translocations of itself or by upstream aberrance of other nodes, augmented disease pathogenesis, promoted tumor cell survival, and out-of-control cell cycling via classical cytoplasmic JAK signaling or the noncanonical nuclear JAK pathway, both of which rewrite the epigenome and prompt the expression of oncogenes.

In this article, we review activating mutations and fusions of JAKs that enhance JAK/STAT phosphorylation and lead to overexpression of STAT target oncogenes in a couple of lymphoid cancerous contexts, canonical JAK/STAT signaling and the nuclear role of JAKs that non-canonically bind to RNA polymerase II and phosphorylate histones [2] or chromatin modifiers [3,4]. We also summarize the effectiveness of JAK-targeting monotherapy and combinational therapy in curing lymphoid cancers, which induce programmed cell death and cell cycle arrest [5].

## 2. JAK Abnormalities

### 2.1. Abnormally Activating JAK Mutations 

JAK1 mutations have been found in adult precursor T acute lymphoblastic leukemia (ATLL, 18%) [6], T-cell prolymphocytic leukemia (T-PLL, V658F, responding well to JAK1-inhibition therapy) [7], cutaneous T-cell lymphoma (CTCL) [8], anaplastic large cell lymphoma (ALCL, 18%) [9,10], plasmablastic lymphoma (PBL,14%) [11], peripheral T-cell lymphoma (PTCL, G1097D) [12] and enteropathy-associated T cell lymphoma (EATL) [13]. JAK2 mutations have been associated with poor prognosis in pediatric B-cell precursor acute lymphoblastic leukemia (BCP-ALL) [14]. Three different JAK2 mutations, R683G, H574R and I682T, were identified in T-cell lymphoblastic lymphoma (T-LBL), and two of these mutations constitutively activated JAK2/STAT signaling. In primary T-LBL samples harboring JAK2 mutations, LMO2 expression was also increased [15]. Moreover, TYK2 heterozygous mutations were discovered in two siblings who developed Epstein–Barr virus (EBV)-associated B-cell lymphoma. Additionally, under 35% of TYK2 deficiency, these patients responded normally to type I interferon (IFN), IL-6, IL-10 and IL-12, whereas they responded abnormally to IL-23 [16]. 

JAK3 mutations have been reported using next generation sequencing in natural killer/T-cell lymphoma (NKTCL) from cohorts in Singapore [17,18,19], Latin America (Mexico, Peru and Argentina) [20], Korea [21,22], Thailand, Japan [23] and France [24], which partly led to the constitutive phosphorylation of JAK3 [18,24], activation of JAK3/STAT signaling [17,20] and interleukin-independent NKTCL cell survival [21]. The allelic ratio of JAK3 mutations ranged from 3% to 35.4% [18,19,20,21,22,24]. The mutation hotspots were mostly in the JAK3 pseudokinase domain and involved exon 13 [18], A572V, A573V [19,24], H583Y, G589D [21] and V722I [24]. In addition, JAK3 mutations were also reported in CTCL (3%) [6,8,25], T-PLL (30%) [26], ATLL (5%) [6,25], epitheliotropic intestinal T-cell lymphoma (EITL, 35%) [27], EATL [13] and ocular adnexal marginal zone lymphomas (OAML) (11%) [28], resulting in the activation of key cell survival pathways, including JAK3/STAT, with some known gain-of-function mutational hotspots. 

Furthermore, some studies have well described alterations affecting one to multiple cell fate-related nodes of the JAK/STAT pathway, including Hodgkin–Reed–Sternberg (HRS)-like “cells of NK phenotype” [29], primary cutaneous γδ T cell lymphoma (PCGDTL) [30], EITL [31], post-transplant lymphoproliferative disorder (LPD) [32] and CTCL [32], part of which led to upregulated JAK phosphorylation and activation. All the JAK mutations mentioned above are summarized in Table 1.

### 2.2. JAK-Associated Gene Translocation

The firstly identified and heavily studied phenomenon of JAK translocation is TEL-JAK2 fusion. This fusion protein was characterized in T-cell acute lymphoblastic leukemia (T-ALL) patients, which constitutively activated JAK2 tyrosine kinase activity, STAT phosphorylation and conferred cytokine-independent T-ALL cell proliferation [33,34]. The chimeric TEL-JAK protein promoted several downstream oncogenic signals, including ERK, SAPK-JNK, P38 [35], PI3K/PKB [36] and SOCS1 [37]. The TEL-JAK2 drove T-cell leukemia development alone [38] and in cooperation with pre-TCR signaling [39] or TEL-ABL fusion protein [40]. This activating TEL-JAK2 translocation was detected in 2 out of 16 T-ALL patient samples studied [15].

Additionally, a three-way t(9;13;16) (p24;q34;p11) chromosome translocation was detected in a cutaneous CD4 positive T-cell lymphoma case, in which JAK2 was fused to a novel gene ATXN2L. This fusion product contained the full ATXN2L protein and the catalytic domain of the JAK2 kinase, leading to constitutive activation of the JAK2/STAT signaling pathway, similar to the TEL-JAK2 chimeric protein [41]. In one case of classical Hodgkin lymphoma (cHL), the t(4;9)(q21;p24) translocation was observed, which resulted in a new oncogenic and enzymatically activated SEC1A-JAK2 fusion protein. Additionally, the fused protein was sensitive to JAK inhibitors [42]. Interestingly, by genetic profiling of breast implant associated anaplastic large cell lymphoma (BIA-ALCL), JAK2 was found to fuse with its downstream node STAT3, and this is also the first reported fusion fact in BIA-ALCL [43]. Utilizing whole-transcriptome sequencing in CD30+ LPD, a fusion involving NPM1 (5q35) and TYK2 (19p13) was observed. The fusion encoded an NPM1-TYK2 chimeric protein containing the oligomerization domain of NPM1 and an intact catalytic domain in TYK2. The NPM1-TYK2 fusions were found in 2 of 47 (4%) primary cases and functionally evoked activation of TYK2 and STAT1/3/5 [44]. A recurrent chimera combining transcription factor NFkB2 and TYK2 was also discovered in WT JAK1/STAT3 ALK(-) ALCL [10]. Moreover, JAK chimeric aberrations were also identified in BCR-ABL1-like pediatric BCP-ALL [14], CTCL [45] and pediatric cHL [46]. 

## 3. JAK Signaling

### 3.1. Upstream Drivers for JAK Activation

This section describes how JAKs are deregulated by kinase/phosphatase, non-cytokine stimulus and trans-modulated by other factors. As members of the class I nonreceptor protein tyrosine phosphatase family, PTPN proteins are ubiquitously expressed with high levels in immune cells [47]. In cHL, splice variants of PTPN1, which missed one or more exon sequence and were catalytically inactive, augmented downstream JAK/STAT signaling [48,49]. As a tumor suppressor capable of inhibiting the JAK/STAT pathway, PTPN2 suppressed T cell proliferation. Therefore, bi-allelically inactivated PTPN2 identified in 2 out of 39 cases of PTCL led to JAK/STAT activation [50]. Similarly, PTPN6 loss-of-function N225K and A550V mutants exhibited reduced tyrosine phosphatase activity and caused the deregulated JAK3/STAT3 pathway in diffused large B cell lymphoma (DLBCL) [51]. Moreover, the PIM serine/threonine kinase aberrant expression and activation appeared in several cancerous contexts, including primary mediastinal large B-cell lymphoma and cHL, promoting cancer cell survival and immune surveillance escape partly via modulating JAK/STAT activity [52,53]. Abnormal suppression of SHP1/2 and SOCS-1 in multiple myeloma (MM) plasma cells significantly correlated with the sustained activation of the JAK/STAT3 pathway [54]. A double kinase fusion ITK-SYK was identified in PTLC, which drove cellular transformation and progression of this malignancy. Additionally, through microarray data analysis, JAK3/STAT5 activation was discovered as a downstream effect of ITK-SYK aberrance, and pharmacological inhibition of JAK3 abrogated STAT5 phosphorylation, suppressed cell survival and induced G1/S phase arrest [5]. 

Several non-cytokine upstream stimuli have been recounted to directly affect JAK/STAT signaling. By exploiting the IL-10/JAK pathway, the human T-cell leukemia virus type 1 (HTLV-1) viral protein HBZ induced an increased IL-10 level, suppressed host immune response and therefore upgraded HTLV-1 proliferation in infected T leukemia cells [55]. In cHL, lymphotoxin-α was characterized as one of the factors that promotes JAK2/STAT6 activation, as dissected by chromatography coupling with mass spectrometry [56]. In MM cells, hypoxia-dependent erythropoietin (EPO)-receptor was shown to be upstream of the JAK signaling pathway. JAK2 could be phosphorylated by recombinant EPO in kinase assay and EPO exposure intriguingly reduced myeloma cell survivals [57].

Trans-mediation of JAK family proteins was also reported in recent years. In high-grade B-cell lymphoma, BCL6 was characterized as a transcription factor, which directly bound to the JAK2 promoter, as evidenced by ChIP-seq [58]. In DLBCL and follicular lymphoma (FL), the histone methyltransferase KMT2D has been shown as a bona fide tumor suppressor and one of the most frequently mutated genes. KMT2D directly mediated histone H3K4 methylation and thereby perturbs expression of a set of genes, including JAK/STAT [59]. miR-155, associated with poor prognosis, has been implicated in the progression of CTCL. This microRNA simultaneously modulated multiple survival-associated pathways, including JAK/STAT. Cobomarsen, a locked nucleic-acid-modified oligonucleotide inhibitor of miR-155, effectively saved expression of these survival cascades [60]. The JAK signaling pathway could be driven by MALT1 [61], MYD88 [62], HSP90 [63] and SOD [64] via undescribed mechanisms. 

### 3.2. Classical JAK/STAT Pathway

The cytokine/JAK/STAT pathway starts when a cytokine binds to its cognate receptor and induces the dimerization and phosphorylation of the receptor on its intracellular domain. These receptors contain a common γ chain and a unique α chain. Specifically, IL-2 and IL-15 receptors share an additional IL-2/IL-15Rβ subunit [1]. The receptor activation further causes JAKs protein phosphorylation, creating docking sites for STATs phosphorylation and dimerization. The dimerized STAT then transfers to the nucleus and trans-regulates gene expression via binding to DNA consensus sequences [65]. 

STAT3, firstly identified in 1993 in a biochemical study, has been the most-studied member within the STAT family [65]. The JAK/STAT3 cascade was mutated and aberrantly activated [10,66,67,68] in a number of lymphoid cancers, rendering cytokine-independent activation [69], immunosuppression- and tumor growth-related gene expressions (MCL1, SOX11, CD38, PD-L1, MUC1, MCL1, MYC and GTPase RhoU) [17,70,71,72,73,74,75,76], sustained tumor cell survival [71], prompted cell migration [76], differentiation advantage towards terminally differentiated B-cell lymphoma [77], resistance to cytotoxic and biological agents [74], disease progression [78] and shorter event-free survival [79]. Moreover, other STAT family members, such as STAT1, STAT5 and STAT6 were also mutated, upregulated, phosphorylated and activated in lymphoid disease subsets [69,71,80,81,82,83,84,85], resulting in increased expression of downstream nodes, such as BATF3 and MYC [86]. The JAK/STAT1/5/6 signaling was enriched in disease cohorts [87,88,89,90,91], which drove pathogenesis [89] and neoangiogenesis [85] and was associated with elevated frequencies of lymphoid malignancies [92]. 

### 3.3. Newly Identified Nuclear JAK Signaling

**Figure 2:**
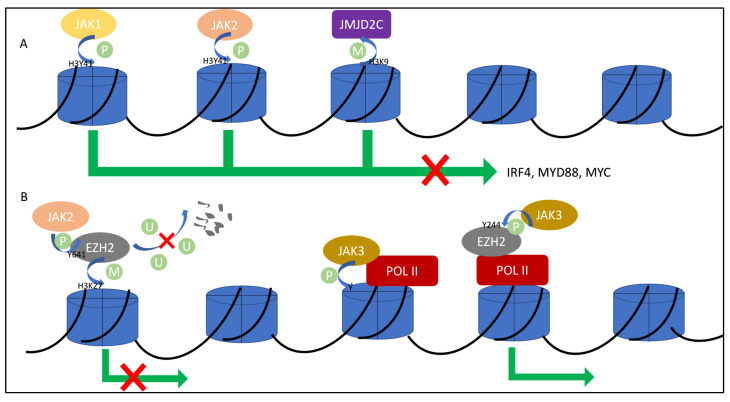
Schematic model showing the nuclear role of JAKs. (**A**) Phosphorylation maintained by JAK1/2 and de-methylation maintained by JMJD2C leads to gene repression. (**B**) JAK2 phosphorylates EZH2 and blocks EZH2 ubiquitination. JAK3 phosphorylates histone and EZH2, therefore promoting gene expression.

In addition to the traditional JAK/STAT signaling cascade, non-STAT phosphorylation and the nuclear role of JAKs have been proposed, which strongly relate to the pathogenesis and progression of lymphomas. In primary mediastinal B cell lymphoma (PMBL) and cHL, JAK2-mediated H3Y41 phosphorylation co-operated with JMJD2C-modulated H3K9 demethylation, thereby silencing the myc oncogene, promoting heterochromatin formation and remodeling epigenome [2] (Figure 2A). The H3Y41 locus may also be phosphorylated by JAK1, thus regulating nearly 3000 proliferation- and survival-associated genes in activated B cell-like diffuse large B cell lymphoma (ABC-DLBCL), including IRF4, MYD88 and MYC [93] (Figure 2A). Nuclear JAK3 has also been observed in CTCL cells, which interacted with the catalytic subunit of RNA polymerase II and phosphorylated histone H3 on its tyrosine residue [94] (Figure 2B). Epigenetic phosphorylation by JAK family members occurs on histone modifiers as well. We have shown that in NKTCL, JAK3 transferred to the nucleus and phosphorylated PRC2 methyltransferase EZH2 at Y244, switching EZH2 from an epigenetic silencer to a transcriptional activator (Figure 2B). The downstream activated genes were related to stemness, invasiveness, DNA replication, cell cycle, oncogenesis and proliferation [3]. Similarly, JAK2 also site-specifically phosphorylated EZH2 at Y641, and rendered EZH2 to avoid β-TRCP-mediated proteosomal degradation [4] (Figure 2B). Apart from JAK-catalyzed phosphorylation, JAK3 and SUZ12 mutations orchestrated to drive T-cell transformation and T-ALL development [95].

## 4. JAK-Based Targeted Therapeutics

### 4.1. Monotherapy

The most widely known JAK inhibitor tested in lymphoma trials is Ruxolitinib. This potent compound selectively inhibits JAK1 and JAK2 and is administrated orally. Ruxolitinib has been approved for the treatment of myelofibrosis (MF) by the US Food and Drug Administration (FDA) in 2011 and by the European Medicines Agency (EMA) in 2012, followed by the approval for treatment of hydroxyurea (HU)-resistant or -intolerant polycythemia vera (PV) in 2014 [96]. The drug is not only specific for the mutated form of JAK2 but also inhibits the wild-type JAK2 [97]. In cHL, Ruxolitinib has been seen to induce anti-proliferative effects and programmed cell death in vitro and significantly inhibited tumor progression and improved survival in vivo [98]. Effects of Ruxolitinib in cHL have also been validated in clinical trials, with a disease control rate of 54% (7/13) and a median response duration of 5.6 months [99], or an overall response rate of 9.4% (3/32) after six cycles of dosing for relapsed/refractory cases [100]. In MM, Ruxolitinib treatment decreased expression of genes including JAK2, TYK2, IL-6 and IL-18, driving disease progression and inducing antophagosome accumulation [101]. In a phase I clinical trial, Ruxolitinib was able to overcome lenalidomide and steroid resistance for relapsed/refractory MM patients, with a clinical benefit rate of 46% and an overall response rate of 38%, respectively [102]. Hypersensitivity of Ruxolitinib was noted in one patient with CSF3R T618I mutation, in which there were decreased white cell numbers and neutrophil counts as well as a normalization of the platelet count [103]. Effectiveness of Ruxolitinib was also seen in primary cutaneous CD8+ aggressive epidermotropic cytotoxic T-cell lymphoma [104], BCP-ALL [14] and ALCL [105], in which the JAK/STAT pathway played a vital role. However, whether Ruxolitinib is effective in treating PMBL remains controversial [98,99]. This medication has been approved to enter clinical trial phase I/II/III for the treatment of lymphoma, lymphoblastic leukemia or MM alone or together with other agents (NCT01877005, NCT01965119, NCT02164500, NCT02974647, NCT03117751, NCT03041636, NCT02723994, NCT03613428, NCT01712659, NCT03878524, NCT01914484, NCT01620216, NCT00674479, NCT00639002 and NCT03773107). The immunosuppressive side effects of Ruxolitinib have been reviewed extensively before [97]. 

Tofacitinib, an oral and small molecule compound, inhibits all four JAKs but preferentially inhibits JAK1 and JAK3 [106]. In EBV+ T and NK lymphoma cell lines and patient samples which displayed JAK3/STAT5 activation, Tofacitinib treatment effectively reduced p-STAT5 levels, suppressed proliferation, induced G1 cell cycle arrest and decreased EBV viro-protein LMP1 and EBNA1 expression [107]. In CTCL cells, Tofacitinib inhibited the level of aberrantly expressed anti-apoptotic miR-21 by blocking JAK3/STAT5 signaling, and STAT5 could directly bind to miR-21 promoter [108]. This drug reversed the majority of pro-survival signals modulated by JAK-STAT cascade in MM [109]. In PTCL, as mentioned above, the JAK3/STAT5 signaling program was identified to be downstream of ITK/SYK via Signal Net and cluster analyses of microarray data. JAK3 selective inhibitor tofacitinib abrogated the phosphorylation of STAT5, suppressed cell growth, induced cell apoptosis and arrested the cell cycle at the G1/S phase [5]. As JAK3-activating mutation was frequent in NKTCL pathogenesis, the pan-JAK inhibitor Tofacitinib efficiently reduced phosphorylated STAT5 and cell viability in JAK3-mutant and wild-type NKTCL cell lines and mouse xenografts [19,24]. However, in one case of relapsed T-ALL with two JAK3 activating mutations, Tofacitinib failed to induce a positive clinical response following failure of salvage chemotherapy, indicating that the presence of activating JAK3 mutations did not necessarily guarantee sensitivity to Tofacitinib treatment [110]. 

Moreover, several JAK-targeting new compounds or derivatives as well as JAK upstream inhibitor have been reported in recent years. Here I summarize these inhibitors based on the types of malignancy. In DLBCL, a natural osalmid derivative DCZ0858 blocked JAK2/STAT3 signaling and inhibited B lymphoma cell survival in a concentration- and time-dependent manner while causing no significant toxicity to normal B cells [111]. Additionally, upstream IRAK4 inhibition by highly selective novel small molecule inhibitors, ND-2158 and ND-2110, impeded survival of DLBCL cells by downregulating survival signals, including IL6/IL10/JAK/STAT3 [112]. In another lethal and skin-attacking lymphoma CTCL, a retinoic acid derivative, ECPIRM, induced cell apoptosis and induced G0/G1 phase arrest via inhibiting the JAK/STAT rather than the RAR/RXR pathway and exhibited little cytotoxicity in normal lymphoid counterparts [113]. Besides, a vitamin A derivative, 9-cis-RA, induced CTCL cellular apoptosis dose- and time-dependently via decreasing JAK1/STAT3/STAT5 phosphorylation, Bcl-xL and cyclin D1 levels [114]. A novel taspine derivate TPD7 was able to bind to the IL-2 receptor in CTCL and therefore suppressed the downstream cascade, including JAK/STAT and PI3K/AKT/mTOR [115] Additionally, another compound ONC201 exerted time-dependent cell survival inhibition in CTCL cell lines and patient-derived primary CD4+ malignant T cells, and the JAK/STAT pathway was downregulated with ONC201 treatment [116]. These derivatives or inhibitors demonstrated effectiveness and selectivity in harnessing JAK/STAT in order to treat CTCL. In NKTCL, frequent STAT3/5B activating mutations were detected in primary patient samples and cell lines, and JAK1/2/3 inhibitors potently suppressed cellular proliferation, inhibited tumor growth and induced apoptosis via abrogation of JAK/STAT program [117,118]. Moreover, NKTCL is known for EBV infection, which is also one of the criteria for NKTCL diagnosis, and LMP1 was a viro- and onco-protein generated by EBV. In NKTCL, a constructed human anti-LMP1 antibody successfully inhibited cell proliferation, induced apoptosis and activated antibody-dependent cell-mediated cytotoxicity and complement-dependent cytotoxicity at least partly via inhibiting JAK3/STAT3 [119]. Even classic cytotoxic agents also exhibited anti-JAK/STAT properties. Doxorubicin inhibited c-myc and PIM1 expression by repressing JAK/STAT3 and promoted NKTCL cell death [120]. In MM, compounds including Icarrin, 3-formylchromone, TM-233, Auranofin, AZD1480, thalidomide analogs and tetracyclic pyridone 6, inhibited upstream JAK1/2, thereby blocking constitutive STAT3 phosphorylation and its nuclear translocation, downregulating downstream STAT3 target genes, such as Bcl-2, Bcl-xl, survivin, COX-2, VEGF, Mcl-1, Cyclin D2 and MMP-9 and inducing programmed cell death [121,122,123,124,125,126,127]. Similarly, two novel and highly selective JAK inhibitors, INCB20 and INCB16562, effectively suppressed IL-6 dependent growth of MM cell lines and primary bone marrow-derived plasma cells [128,129]. In addition, several natural product extracts blocked JAK/STAT as well and exerted anti-myeloma effects. Leelamine from pine’s bark attenuated phosphorylation of upstream JAK1/JAK2/Scr macromolecules and downstream STAT3, hence evoking myeloma cell cycle arrest and apoptosis [130]. A Scutellaria radix component, Baicalein, suppressed myeloma cell survival and proliferation by blocking IκB-α degradation, followed by downregulating IL-6/JAK/STAT3 and XIAP gene levels [131]. These findings demonstrated possibilities to inhibit myeloma cell survival, proliferation and invasiveness via targeting JAK/STAT using synthesized compounds and natural extracts. Moreover, in waldenström macroglobulinemia (WM), the pan-FGF trap molecule NSC12 significantly inhibited cellular growth and provoked apoptosis through halting JAK/STAT3, MAPK and PI3K-AKT pathways [132]. All the JAK-based monotherapies are summarized in Table 2.

### 4.2. Combinational Therapy

The most heavily studied and JAK-related dual inhibitor should be Cerdulatinib. This orally available compound demonstrates activities against JAK1/3 and SYK with limited inhibition of JAK2. Cerdulatinib did not inhibit phorbol-mediated signaling or activation in normal B and T cells, or T-cell receptor mediated signaling in T cells, showing selectivity and safety [133]. This inhibitor exerted potent antitumor activities in a subset of B-cell lymphomas, including ABC-DLBCL, germinal center-diffuse large B cell lymphoma (GC-DLBCL), mantle cell lymphoma (MCL), FL and small lymphocytic lymphoma (SLL) [133,134,135]. In CLL, the dual JAK/SYK inhibitor Cerdulatinib was a promising therapeutic agent that overcame the support of the microenvironment [136] and targeted critical survival pathways, used either alone or combined with Venetoclax [137]. This compound also displayed efficacies in ATLL [138]. Activities of Cerdulatinib against lymphoid tumors were evaluated in clinical trial phase I/II (NCT01994382 and NCT04757259). Another notable JAK-associated dual inhibitor is SB1518, which co-targets JAK2 and FLT3. This compound was selected as a development candidate and progressed into clinical trials for lymphomas [139]. SB1518 demonstrated safety and efficacy in various types of lymphomas, including refractory cases, and a phase I clinical trial demonstrated that an escalating dose of SB1518 led to significant tumor reduction of 4–46% among enrolled patients of relapsed/refractory lymphomas with well-tolerated toxicities [140,141] (NCT01263899 and NCT00741871). 

The most widely known JAK inhibitor, Ruxolitinib, as mentioned above, has been applied in synergism with several different compounds. In ABC-DLBCL, JAK1/STAT3 was activated by autocrine IL-6/10 signaling, and Ruxolitinib synergized well with type I IFN inducer lenalidomide in vitro and in vivo [142]. In MM, both JAK1 and JAK2 presented overexpression in a proportion of patients, and Ruxolitinib treatment in combination with Bortezomib, Itacitinib or Daratumumab inhibited JAK/STAT3 phosphorylation, upregulated CD38 expression, inhibited in vitro and in vivo myeloma cell growth and induced cell apoptosis and subG0 arrest [73,143,144]. In NKTCL, Ruxolitinib and CDK4/6 inhibitor LEE011 treatment demonstrated synergistic growth inhibitory effects [145]. Ruxolitinib and Bcl-2/Bcl-xl inhibitor Navitoclax well synergized with each other, augmenting the expression of Bik, puma and Bax expression in cHL cells [146], lowering tumor burden and prolonging survival in an ATLL mouse model [147]. In CTCL cell lines, Ruxolitinib and Resminostat (HDAC inhibition) together exhibited substantial anti-cancer effects [148]. In relapsed/refractory T-ALL, Ruxolitinib and Venetoclax treatment reduced cell survival and proliferation in vitro [149].

The combination between JAK inhibitor and PI3K inhibitor showed significance in a few lymphoid malignancies. In relapsed/refractory B cell lymphoma, JAK1 inhibitor itacitinib+ PI3Kδ inhibitor INCB040093 demonstrated efficacy and few toxicities, presenting a promising treatment option [150]. In MM, JAK2 inhibitor TG101209 and PI3K inhibitor LY194002 combination displayed synergistic cytotoxicity against myeloma cells [151]. In PI3K inhibitor-resistant B-cell and T-cell lymphoma cell lines, the addition of JAK inhibitor BSK805 circumvented well with PI3K inhibitor acquired resistance in lymphomas, and simultaneous inhibition of these two pathways produced combined effects [152]. 

Successful combinations were also observed for inhibitors against JAK and BTK, a major target for B-cell malignancies [153]. The bromodomain and extra-terminal (BET) inhibitor OTX015 targeted different pathways including JAK/STAT in mature B-cell lymphoid cancer cell lines, and it presented in vitro synergism with BTK inhibitor [154]. The JAK/STAT inhibitor + BTK inhibitor Ibrutinib in combination bypassed survival stimuli from bone marrow mesenchymal stromal cells to induce cell death in CLL [155] and induced IRF4 levels to synergistically kill ABC-DLBCL cells [93].

A couple of studies have evaluated the combination between JAK inhibitors and the anti-apoptotic macromolecule BCL inhibitors. Combined inhibition of JAK and BCL2 demonstrated strong potentiation of cytotoxicity in CTCL cells, driven by intrinsic and extrinsic apoptosis pathways [156]. In Burkitt lymphoma (BL), BCL6 deficiency induced JAK2 expression and STAT3 phosphorylation, and a JAK2 inhibitor, Lestaurtinib, repressed survival of BCL6-deficient cells and tumor xenografts, demonstrating the significance of co-suppressing BCL6 and JAK2, which was considered as synthetic lethality [58]. In cHL, Decitabine inhibited cell growth but concurrently upregulated pro-survival signals, such as MEK/ERK, JAK/STAT and NF-κB, demonstrating a rationale for combining Decitabine with BCL/BCL2L1 inhibitor ABT263, JAK-STAT inhibitors Fedratinib and SH-4-54, AKT inhibitor KP372-1, NF-κB inhibitor QNZ, as well as the BET family proteins inhibitor JQ1 [157].

Investigators also tried to combine JAK inhibitor with conventional therapies in order to ameliorate clinical outcomes. In MCL, anti-JAK/STAT3 agent Degrasyn was considered as a useful therapy administered together with Bortezomib [158]. In MM, selective JAK1 inhibitor INCB052793 in combination with carfilzomib, bortezomib, dexamethasone or lenalidomide effectively reduced tumor volume in tumor-bearing mice [159]; another novel and orally available JAK1/2 inhibitor, CYT387, was able to prevent IL-6-induced STAT3 phosphorylation and was synergized in killing myeloma cells with traditional therapies Melphalan and Bortezomib [160]. JAK inhibitors combined with the cytotoxic anti-folic-acid agent methotrexate significantly suppressed lymphoma cell growth and prolonged survival of tumor xenografts, resulting in better clinical outcomes [161,162]. In CML, targeting JAK/STAT3 cascade by JAK inhibitor in combination with classical BCR-ABL inhibitor promoted cell death and eliminated minimal residual disease located in the bone marrow, representing a hopeful therapeutic strategy [163,164]. 

In addition, as JAK/STAT3 mutations promoted STAT3-based transcription activation and directly regulated NF-κB and CD30 levels in NIK+/ALK- ALCL, combined NIK and JAK inhibitor therapy could be applied to benefit patients [165]. JAK inhibitor AZD1480 treatment potently blocked STAT phosphorylation but yielded no anti-proliferative effects in cHL, as it led to ERK1/2 phosphorylation upregulation. Therefore, inhibiting ERK activities by MEK inhibitors along with JAK inhibition resulted in enhanced cyto-toxicities [166]. Histone deacetylase (HDAC) inhibitors represent an encouraging class of antitumor therapies, and these inhibitors induce minimal toxicity to normal cells [167]. The orally administered HDAC6 inhibitor Citarinostat was used together with JAK/STAT3 inhibitor Momelotinib, resulting in reduced mitochondrial membrane potential, decreased Bcl-2 and Bcl-xl and activated caspase 3/9, indicating extrinsic apoptosis [167]. In Sézary syndrome, an aggressive and diffused form of CTCL, the HDAC inhibitor Romidepsin showed remarkable but transient activity, and the add-in of JAK inhibitor in combination led to markedly increased therapeutic responses [168]. In LPD, constitutive JAK/STAT3 significantly contributed to disease progression, and combinations including JAK, HSP90 and mTOR inhibitors yielded satisfactory effects on repressing cell viability [169]. All the JAK-based combinational therapies are summarized in Table 3.

## 5. Conclusions and Future Directions

Accumulating evidence in this review demonstrates how JAKs are aberrantly expressed in lymphoid cancerous contexts and how JAKs connect with upstream and downstream signaling. JAK abnormalities, either mutation or translocation, were found in a few but not all cases in a variety of lymphoid cancers. These abnormalities augment the signals of the cytokine/JAK/STAT pathways, but do not necessarily support lymphoid tumor survival. In a majority of contexts, JAKs signal through STAT-based activation and transcriptional regulation, whereas in a few contexts, the tyrosine kinase JAKs may phosphorylate histone H3 or EZH2 and reprogram transcription profiles [3,4,93,94]. These findings contribute to the importance of the nuclear role of JAKs.

In the recent decade, a couple of specific small-molecule JAKs inhibitors have been developed and utilized to target JAK abnormalities in lymphoid malignancies, such as Ruxolitinib and Tofacitinib. Ruxolitinib has entered more than 10 clinical trials for lymphoid disease treatment. Several natural product derivatives and traditional medications have also been reported to be able to block JAK/STAT signaling and impede cancer cell survival [111,124]. Combinational JAK inhibition, either through a dual inhibitor or through several agents, exhibits better cell killing effects than monotherapy. These results demonstrate an indispensable role of JAK-targeting in treating lymphoid cancers, and future studies are needed to compare the effects of these JAK inhibition therapies over conventional therapeutics.

## Figures and Tables

**Table 1:** JAK mutations in lymphoid cancers.

Malignancy	Protein(s)	Mutation Site(s)	References
ATLL	JAK3	--	[6]
T-PLL	JAK1	V658F	[7]
CTCL	JAK1	Y654F, L710V	[8]
ALCL	JAK1	R174 *, G1097D/S	[9,10]
PBL	JAK1	G1097D/V	[11]
PTCL	JAK1	G1097D	[12]
EATL	JAK1	--	[13]
Pediatric BCP-ALL	JAK2	R873N, R683T/G/S, D873N, T875N, R923H, K914E	[14]
T-LBL	JAK2	H574R, R683G, I682T	[15]
EBV-associated B-cell lymphoma	TYK2	R231W	[16]
NKTCL	JAK3	M511I, A572V, A573V, H583Y, G589D, R657Q, V722I,	[17,18,19,20,21,22,23,24]
CTCL	JAK3	S989I, Y1023H	[8,25]
T-PLL	JAK3	M511I, A657G, G491S, V674A, V678L, A573V, G507P	[26]
EITL	JAK3	V674A, M511I	[27]
EATL	JAK3	--	[13]
OAML	JAK3	--	[28]
PCGDTL	JAK3	R657W	[30]
EITL	JAK1, JAK3	JAK1: L1026G, S703I, L783P; JAK3: M511I, A573V, V674A	[31]

Abbreviations: ATLL: adult precursor T acute lymphoblastic leukemia, T-PLL: T-cell prolymphocytic leukemia, CTCL: cutaneous T-cell lymphoma, ALCL: anaplastic large cell lymphoma, PBL: plasmablastic lymphoma, PTCL: peripheral T-cell lymphoma, EATL: enteropathy-associated T cell lymphoma, BCP-ALL: B-cell precursor acute lymphoblastic leukemia, T-LBL: T-cell lymphoblastic lymphoma, EBV: Epstein–Barr virus, NKTCL: natural killer/T-cell lymphoma, EITL: epitheliotropic intestinal T-cell lymphoma, OAML: ocular adnexal marginal zone lymphomas, HRS: Hodgkin–Reed–Sternberg, PCGDTL: primary cutaneous γδ T cell lymphoma, LPD: lymphoproliferative disorder. * indicated mutated amino acid not identified.

**Table 2:** JAK inhibition in lymphoid malignancies.

Inhibitor(s)	Malignancies	Mechanism of Action	References
Ruxolitinib	MPN, HL, PMBL, MM, RRMM, CNL, pcAECyTCL, BCP-ALL, ALCL	Inhibit JAK1/2	[14,96,97,98,99,100,101,102,103,104,105]
Tofacitinib	EBV-associated T and NK cell lymphoma, CTCL, MM, PTCL, NKTCL	Inhibit JAK1/3	[5,19,24,107,108,109]
DCZ0858	DLBCL	Inhibit JAK2/STAT3	[111]
ND-2158 and ND-2110	DLBCL	Inhibit IRAK4	[112]
ECPIRM	CTCL	Inhibit JAK/STAT	[113]
9-cis-retinoic acid	CTCL	Inhibit JAK1/STAT3/5	[114]
TPD7	CTCL	Bind to IL-2 receptor	[115]
ONC201	CTLC	Inhibit JAK/STAT	[116]
PRN371	NKTCL	Inhibit JAK3/STAT	[117]
AZD1480	NKTCL	Inhibit JAK1/2	[118]
LMP1-IgG	ENKTL	Inhibit JAK3/STAT3	[119]
Doxorubicin	TCL	Inhibit JAK/STAT3	[120]
Icariin	MM	Inhibit JAK1/2/STAT3	[121]
3-Formylchromone	MM	Inhibit JAK1/2/STAT3	[122]
TM-233	MM	Inhibit JAK2/STAT3	[123]
Auranofin	MM	Inhibit JAK2/STAT3	[124]
AZD1480	MM	Inhibit JAK2/STAT3	[125]
CPS11/CPS49	MM	Inhibit JAK/STAT	[126]
Pyridone 6	MM	Inhibit JAK/STAT	[127]
INCB20	MM	Inhibit JAKs	[128]
INCB16562	MM	Inhibit JAKs	[129]
leelamine	MM	Inhibit JAK1/2	[130]
Baicalein	MM	Inhibit IL6/JAK/STAT3	[131]
NSC12	WM	Inhibit JAK/STAT3	[132]

Abbreviations: MPN: myeloproliferative neoplasm, HL: Hodgkin lymphoma, PMBL: primary mediastinal B-cell lymphoma, MM: multiple myeloma; RRMM: relapsed/refractory multiple myeloma, CNL: chronic neutrophilic leukemia, pcAECyTCL: primary cutaneous CD8+ aggressive epidermotropic cytotoxic T-cell lymphoma, BCP-ALL: B-cell precursor acute lymphoblastic leukemia, ALCL: anaplastic large cell lymphomas, CTCL: cutaneous T cell lymphomas, PTCL: peripheral T-cell lymphoma, NKTCL: natural killer/T-cell lymphoma, DLBCL: diffuse large B-cell lymphoma, ENKTL: extranodal nasal-type natural killer (NK)/T-cell lymphoma, TCL: T-cell lymphomas, WM: waldenström macroglobulinemia.

**Table 3:** Combining JAK inhibitors with other chemo-agents in lymphoid malignancies.

Regimen	Malignancies	Mechanism of Action	References
Cerdulatinib	B-cell malignancies, ABC-DLBCL, GC-DLBCL, MCL, FL, SLL, CLL, ATLL	Inhibit JAK1/3 and SYK	[133,134,135,136,138]
Cerdulatinib and Venetoclax	CLL	Inhibit JAK1/3, SYK and Bcl-2	[137]
SB1518	Relapsed/refractory lymphoma	Inhibit JAK2 and FLT3	[139,140,141]
Ruxolitinib and Lenalidomide	ABC-DLBCL	Inhibit JAK1/2 and induce type I IFN	[142]
Ruxolitinib and Daratumumab	MM	Inhibit JAK1/2 and upregulate CD38	[73]
Ruxolitinib, Bortezomib and Itacitinib	MM	Inhibit JAK1/2 and proteasome	[143]
INCB054329 and Ruxolitinib/Itacitinib	MM	Inhibit JAK1/2 and BET	[144]
Ruxolitinib and LEE011	NKTCL	Inhibit JAK1/2 and CDK4/6	[145]
Brentuximab Vedotin and Ruxolitinib/Navitoclax	HL	Inhibit JAK1/2 and Bcl-2/Bcl-x, anti-CD30	[146]
Ruxolitinib and Navitoclax	ATL	Inhibit JAK1/2 and Bcl-2/Bcl-xl	[147]
Ruxolitinib and Resminostat	CTCL	Inhibit JAK1/2 and HDAC1/3/6	[148]
Ruxolitinib and Venetoclax	Relapsed/refractory T-ALL	Inhibit JAK1/2 and Bcl-2	[149]
Itacitinib and INCB040093	Relapsed/refractory BCL	Inhibit JAK1 and PI3Kδ	[150]
TG101209 and LY194002	MM	Inhibit JAK2 and PI3K	[151]
BSK805 and Copanlisib/Duvelisib	B-cell and T-cell lymphoma	Inhibit JAKs and PI3K	[152]
Ibrutinib and OTX015	B cell lymphoma	Inhibit JAK/STAT and BTK	[154]
Ibrutinib and AG490/Stattic	CLL	Inhibit JAK/STAT and BTK	[155]
Ibrutinib and AZD1480	ABC-DLBC	Inhibit JAK2 and BTK	[93]
Ruxolitinib and Venetoclax	CTCL	Inhibit JAK1/2 and Bcl-2	[156]
ABT263/Fedratinib/SH-4-54/KP372-1/QNZ/JQ1 and Decitabine	cHL	Inhibit JAK/STAT, BCL/BCL2L1, NFκB, AKT and BET	[157]
Degrasyn and Bortezomib	MCL	Inhibit JAK/STAT3 and proteasome	[158]
Carfilzomib/Bortezomib/Dexamethasone/Lenalidomide and INCB052793	MM	Inhibit JAK1 and proteasome, induce type I IFN	[159]
CYT387 and Melphalan/Bortezomib	MM	Inhibit JAK1/2 and proteasome	[160]
Antcin H and Methotrexate	BCL	Inhibit JAK and folic acid	[161]
csDMARDs and Methotrexate	NSHL, AML	Inhibit JAK and folic acid	[162]
Nilotinib and INC424	CML	Inhibit JAK and Bcl-Abl	[163]
INK inhibitor and JAK inhibitor	ALCL	Inhibit JAK and INK	[165]
AZD1480 and UO126/PD98059	HL	Inhibit JAK and MEK	[166]
Citarinostat and Momelotinib	Lymphoid malignancies	Inhibit JAK/STAT3 and HDAC6	[167]
Romidepsin and Mechlorethamine	CTCL	Inhibit JAK and HDAC	[168]
INK128/Temsirolimus/Ruxolitinib and Luminespib	LPD	Inhibit JAK/STAT3, HSP90 and mTOR	[169]

Abbreviations: ABC-DLBCL: activated B cell-like diffuse large B cell lymphoma, GC-DLBCL: germinal center-diffuse large B cell lymphoma, MCL: mantle cell lymphoma, FL: follicular lymphoma, SLL: small lymphocytic lymphoma, CLL: chronic lymphocytic leukemia, ATLL: adult precursor T acute lymphoblastic leukemia, MM: multiple myeloma, BCL: B cell lymphoma, NKTCL: natural killer/T-cell lymphoma, HL: Hodgkin lymphoma, ATL: adult T-cell leukemia, CTCL: cutaneous T-cell lymphoma, T-ALL: T cell acute lymphoblastic leukemia, cHL: classical Hodgkin lymphoma, MCL: mantle cell lymphoma, NSHL: nodular sclerosis Hodgkin’s lymphoma, AML: acute myeloid leukemia, CML: chronic myeloid leukemia, ALCL: anaplastic large cell lymphomas, LPD: lymphoproliferative disorder.
